# Is accuracy enough? trust and barriers to AI-based clinical decision support in clinical neurophysiology

**DOI:** 10.1016/j.cnp.2026.04.003

**Published:** 2026-04-07

**Authors:** Øystein Dunker, Leonora Onarheim Bergsjø, Gro-Hilde Severinsen, Henrik Glette, Kristian Bernhard Nilsen

**Affiliations:** a Department of Neurology, Division of Clinical Neuroscience, Oslo University Hospital, Oslo, Norway; b Department of Natural Sciences, Practical-Aesthetic, Social and Religious Studies, Faculty of Teacher Education and Languages, Østfold University College, Norway; c Centre for e-health/Department of Health and Nursing Science, Faculty of Health and Sport Sciences, University of Agder, Norway; d Norwegian center for e-health research, Tromsø, Norway; e Norwegian Brain Council, Oslo, Norway; f Institute of Clinical Medicine, Faculty of Medicine, University of Oslo, Norway

**Keywords:** Artificial intelligence, Clinical decision support system, Trust, Attitudes

## Abstract

**Objective:**

To investigate attitudes, trust, expectations and concerns among patients and healthcare personnel regarding the use of AI-based clinical decision support systems (AI-CDSS) in clinical neurophysiology.

**Methods:**

We iteratively designed a questionnaire and conducted a national survey of patients and healthcare personnel in Norway. Patients were recruited from a quality registry, while healthcare personnel and technicians were recruited from hospital laboratories. Items included binary and Likert-scale questions addressing attitudes, expectations, concerns and trust, as well as a scenario-based trust assessment. Between-group comparisons were performed using non-parametric and categorical statistical tests.

**Results:**

Both groups reported positive attitudes toward AI-CDSS. Patients more often expected AI-CDSS to improve diagnostic accuracy (p = 0.005), while healthcare personnel anticipated improved equity of services across regions (p = 0.017). Key concerns in both groups were related to responsibility, doctor-patient relationships, AI literacy, overreliance on AI, and potential deskilling. The scenario-based question revealed lower trust when AI-CDSS directly influenced decision-making without physician involvement. Trust in public institutions was high, whereas trust in commercial or industry use of health data was consistently low.

**Conclusions:**

Attitudes toward AI-CDSS in clinical neurophysiology are positive, but diagnostic accuracy isn’t enough: Acceptance is conditional on human oversight, delineated responsibilities, and steps taken to improve transparency and minimize deskilling. Patients want to share health data for AI purposes, but the commercial sector needs to build trust.

**Significance:**

AI-CDSS in clinical neurophysiology is promising, but design and implementation need to address key concerns shared by both patients and health care personnel.

## Highlights

• Patients and clinicians expect AI to improve efficiency and accuracy.
• Key concerns: responsibility, overreliance, AI literacy, doctor–patient relationship.
• Respondents with high institutional trust, but low trust in commercial AI companies.

## 1 Introduction

Today’s healthcare systems are increasingly challenged by an ageing population, a rising burden of disease, and growing expectations for appropriate and timely services. In this context, the use of artificial intelligence (AI) to enhance efficiency and improve patient care has emerged as an appealing prospect in many fields, including clinical neurophysiology. Although definitions of AI systems vary, it has been succinctly described as “the discipline that creates computer systems capable of activities normally associated with cognitive effort” (Solomonides et al., 2022)..

AI systems have the potential to improve diagnostic and prognostic accuracy, increase efficiency, support clinical documentation, reduce the risk of human error, provide (real-time) decision support, and ultimately enhance patient safety, health literacy, fairness, equity, sustainability and quality of care (Al Kuwaiti et al., 2023, Alowais et al., 2023, Ayorinde et al., 2024, Balakrishnan et al., 2025, Elhaddad and Hamam, 2024, Gholipour et al., 2023, Ray et al., 2022, Scheetz et al., 2021, Wang et al., 2023). At the same time, there are clear risks associated with utilizing AI systems, for instance related to transparency, responsibility, doctor-patient relationships, and de-skilling (MIT, 2026). In 2018, the first AI-based diagnostic device was approved by the U.S. Food and Drug Administration (FDA), which incorporated an adaptive algorithm to screen ophthalmic images for retinal diseases or conditions (FDA, 2018). Following this was an explosion in FDA-approved AI-based devices and algorithms for healthcare, predominantly within radiology and cardiology (Benjamens et al., 2020), but also within neurology, including systems such as Ensosleep for the diagnosis of sleep disorders (FDA, 2021) or BrainScope for structural brain assessment after head injury (FDA, 2014).

The ability to train AI on vast amounts of complex health data is particularly relevant to clinical neurophysiology, a field well suited to leverage advances in data-driven methods in general and AI in particular (de Jonge et al., 2024, Ray et al., 2022). Clinical neurophysiological practice provides ample access to rich, high-dimensional raw data, often accompanied by detailed metadata, labels and annotations, while simultaneously working toward increased standardization of methodologies and data formats (Berry et al., 2012, Halford et al., 2021, Stålberg et al., 2019). Moreover, AI-based support can address several inherent challenges in the field, such as variable interrater reliability and need for expert interpretation (Husain, 2025). AI has already shown great potential to improve the interpretation of electromyography (de Jonge et al., 2024, Ray et al., 2022), nerve conduction studies (Öten et al., 2024), polysomnography (Thapa et al., 2026) and electroencephalography (AbuAlrob et al., 2025, Han et al., 2024, Ray et al., 2022), with one such system already receiving FDA approval for discriminating between epileptiform and non-epileptiform routine EEGs (Tveit et al., 2023).

However, developing effective AI systems is one thing, and successful implementation quite another (NOU4, 2023, Severinsen et al., 2025, Silsand et al., 2024). Amongst barriers to implementation of AI systems are the users’ and patients’ attitudes, trust, expectations and concerns. In general, perceptions and attitudes toward AI in healthcare are positive amongst healthcare personnel, healthcare students and patients (Al Hadithy et al., 2023, Amiri et al., 2024, Fritsch et al., 2022, Mousavi Baigi et al., 2023, Oh et al., 2019, Sandanasamy et al., 2025, Stewart et al., 2024, Wang et al., 2023, Young et al., 2021), while healthcare personnel’s trust in AI systems’ accuracy and judgments remains mixed (Ayorinde et al., 2024). Several studies show that patients prefer human physicians over AI, even if the performance of the AI system is non-inferior or even superior (Fritsch et al., 2022, Longoni et al., 2019, Yokoi et al., 2021, York et al., 2020). In general, people tend to quickly lose trust in AI algorithms after even making one single error, while humans are afforded more contextual interpretation of mistakes (Dietvorst et al., 2015). This so-called *algorithm aversion* exemplifies a trust-calibration barrier that must be addressed before AI systems can be successfully implemented in clinical neurophysiology practice.

There is a growing literature on barriers to implementation of AI in healthcare in general, but findings can highly depend on factors such as medical field, care setting, geographical dispersion of health services, and levels of institutional trust. Therefore, we investigated attitudes, trust, expectations, and concerns related to the use of AI in clinical neurophysiology specifically, within a healthcare system characterized by regional disparities and a high degree of trust in strong public institutions (DFØ, 2026).

## 2 Methods

To explore the attitudes, trust, expectations and concerns among users and patients toward implementation of AI-based clinical decision support systems (AI-CDSS) within the field of clinical neurophysiology, we conducted a cross-sectional survey using the below-described questionnaire. The questionnaire was coded in a universally designed self-service online form (Nettskjema, University of Oslo), that could be completed on any device; PC, tablet, and mobile phone.

The study was funded by the Foundation Dam, grant number SDAM_UTV532216. Data is available to researchers upon reasonable request.

### 2.1 Development of questionnaire

The questionnaire was developed and iteratively refined by the authors, and one patient representative. First, we conducted a selective literature review of studies and created a list of items addressing related themes (Al Hadithy et al., 2023, Amann et al., 2023, Beets et al., 2023, Fritsch et al., 2022, Giavina-Bianchi et al., 2024, Katirai et al., 2023, Kostick-Quenet et al., 2024, Mousavi Baigi et al., 2023, Rojas et al., 2022, Shevtsova et al., 2024, Steerling et al., 2023, Stewart et al., 2024, Yoo et al., 2023, Young et al., 2021). These were filtered and adapted to ensure relevance and meaningful content within clinical neurophysiology. Second, additional items were developed to address constructs not sufficiently covered in the existing literature, including domain-specific aspects of clinical neurophysiology and the four predefined domains of interest (attitudes, expectations, concerns, and trust). All proposed items were discussed within the group and the ones judged to have high face validity were included in the first draft.

To promote consistency, ease of completion, and comparability across items, the questionnaire primarily employed closed-ended response formats, using either binary (yes/no) responses or a five-point Likert scale (e.g., disagree, slightly disagree, neutral/not important to me, slightly agree, agree). In addition, optional open-text fields were included to allow respondents to elaborate on expectations, concerns, and factors perceived to increase or decrease their trust in AI-CDSS. A formal qualitative analysis of the text was not performed, as this would require a distinct methodological approach beyond the scope of the present study. However, selected recurring themes from the free-text responses are summarized descriptively in the Results section to provide additional context to the quantitative findings.

A short scenario describing a plausible clinical use case was included early in the questionnaire to make the concepts less abstract and to frame subsequent questions. This scenario was intended to contextualize the implementation of AI-CDSS within clinical neurophysiology practice. In the scenario, a nurse technician conducts a standardized clinical neurophysiological assessment of suspected carpal tunnel syndrome. The nurse technician then uses an AI-CDSS, described as having diagnostic accuracy equivalent to that of a physician, and determines that the patient does not need to see a specialist physician. Respondents were then asked to rate their agreement with the statement: *“I would have equal trust in the decision from the AI system as from a physician/myself (if physician respondent).”*.

An introductory page was also included that defined AI as “digital systems that perform tasks that would normally require human intelligence”. It also provided a healthcare-related example, noting that AI-CDSS for fracture recognition have recently been implemented in some Norwegian hospitals (Severinsen et al., 2025, Silsand et al., 2024). Beyond this, minimal additional information was provided in order to avoid influencing respondents’ baseline attitudes, knowledge, expectations, or concerns.

A pilot study was conducted with 20 representative respondents, including healthcare personnel and neurophysiology technicians from Norwegian clinical neurophysiology departments, patients and user representatives, and researchers in health– and implementation science. The pilot assessed clarity and consistency of question interpretation, relevance and completeness of content, response burden (target completion time ≤ 5 min), and technical functionality across devices (PC, tablet, and mobile phone). Revisions were made following the pilot study based on feedback. No formal psychometric validation or assessment of reliability was performed, as the questionnaire was designed for exploratory purposes.

### 2.2 Cohort and data collection

Patients were recruited from the Neuroscience Registry at Oslo University Hospital, a medical quality registry established for research and quality assurance in clinical neurophysiological practice. The patients had previously been examined at the Section for clinical neurophysiology at Oslo University Hospital and signed a broad informed consent allowing their data to be stored in the neuroscience registry for future research, and permitting future contact for research purposes. We recruited this cohort to ensure inclusion of patients that are in direct contact with the healthcare system and have experience with clinical neurophysiology examinations, enabling them to meaningfully relate to the questionnaire items. Invitation with link to the questionnaire was sent by SMS, with one reminder sent to non-responders after two weeks.

Healthcare personnel and neurophysiology technicians were recruited from clinical neurophysiology laboratories across Norway. The questionnaire link and QR code, accompanied by brief information about the study were distributed by the authors to department- or section leaders. The recipients were then asked to either forward the invitation to their staff, or to present the study during internal meetings, with time allocated for questionnaire completion.

There were no other inclusion or exclusion criteria beyond belonging to one of the two target groups. Data was collected between May and August 2025. Responses were collected voluntarily and anonymously. Due to the distribution model, response rates among healthcare personnel and neurophysiology technicians could not be determined.

### 2.3 Statistics

Statistical analyses were performed using SPSS version 25 (IBM Corp., Armonk, NY, USA). All questionnaire items were set as mandatory, and therefore no missing data was present and no imputation was required. All submitted questionnaires were included in the analyses.

Between-group comparisons were conducted using independent samples t-tests for continuous variables; Chi-square or Fisher’s exact tests for categorical variables; and Mann–Whitney-U tests for ordinal (Likert) variables transformed to 1–5. Group comparisons for the scenario item with three independent groups were performed using the Kruskal–Wallis test: if statistically significant, post-hoc pairwise comparisons were conducted using Mann–Whitney-U tests.

All statistical tests were two-tailed. A p-value < 0.05 was considered statistically significant. Adjustment for multiple comparisons was not performed, as the questionnaire items were designed to address distinct constructs and analyses were considered exploratory.

## 3 Results

The questionnaire was sent out to 1204 patients and to all healthcare personnel and neurophysiology technicians (hereafter healthcare personnel for brevity) employed at clinical neurophysiology laboratories in Norwegian hospitals. There were 193 responses in total, consisting of 129 patients (11% of recipients) and 64 healthcare personnel (unknown %). Sample characteristics are presented in Table 1.

**Table 1:** Sample characteristics.

*n = 193*		Healthcare personnel and neurophysiology technicians	Patients
n (% of total sample)		64 (33)	129 (67)
Age, years (SD)		49 (11)	54 (15)*
Female, n (%)		44 (69)	60 (47)*
Education, n (%)			
	*Primary school*	0 (0)	8 (7)*
	*High school*	2 (3)	30 (24)*
	*1*–*3 years of higher education*	22 (34)	41 (31)
	*4 + years of higher education*	40 (63)	50 (38)*
Title, n (%)			
	*Physician*	22 (34)	−
	*Nurse*	23 (36)	−
	*Other*	19 (30)	−
Employed as a neurophysiology technician, n (%)		32 (50)	−
Health barometer, mean (SD)**		−	57 (22)
Healthcare utilization, last 3 months, n (%)			
	*0 times*	−	15 (11)
	*1 time*	−	20 (15)
	*2*–*4 times*	−	56 (43)
	*5 + times*	−	40 (31)

*p-value < 0.05; **How good do you consider your own health to be (0 to 100)?

Both healthcare personnel and patients reported to be equally interested in AI (median 4, IQR 1; median 4, IQR 2; p = 0.131); were equally frequent users of AI-based tools such as ChatGPT, Microsoft Co-Pilot or image generation models (median 3, IQR 1; median 3, IQR 3; p = 0.327), and were generally positive towards the use of AI in clinical neurophysiology practice (median 4, IQR 1; median 4, IQR 2; p = 0.235).

A summary of responses to the yes/no items is presented in Table 2, illustrating expectations, concerns, and trust related to the implementation AI-CDSS in clinical neurophysiology, including attitudes toward the use of personal health data for AI development. Patients more frequently expected that AI would improve diagnostic accuracy (p = 0.005), whereas healthcare personnel more often expected AI to contribute to increased inter-regional equality of health services (p = 0.017).

**Table 2:** Expectations, concerns, and trust related to the implementation of AI-based clinical decision support systems in clinical neurophysiology.

	Healthcare personnel and neurophysiology technicians (n = 64)Yes, %	Patients (n = 129) Yes, %	p-value
**When introducing AI in clinical neurophysiology, I expect:**			
Increased efficiency in healthcare	76	64	0.101
Earlier diagnostics	50	51	1.000
More accurate diagnostics	45	67	0.005*
More accessible healthcare services / shorter waiting times	77	66	0.139
Greater equality in healthcare services between regions	48	30	0.017*
None of the above	2	9	0.064
A more efficient workday (with the possibility of handling more patient appointments per day)	69	−	
Cost savings at the department	50	−	
Positive changes in work tasks	59	−	
**When introducing AI in clinical neurophysiology, I am concerned about (descending order):**			
That healthcare personnel rely too much on responses from the AI system	−	65	
That I cannot speak with a doctor when I feel I need to	−	65	
Who is responsible when something goes wrong	−	54	
That healthcare personnel do not have sufficient understanding of how the AI system works	−	50	
That healthcare personnel cannot override errors in the AI system	−	36	
Data security (e.g., personal data may be exposed or the system may be hacked)		34	
That the AI system discriminates because it has been trained incorrectly	−	31	
That AI is still worse than a doctor	−	26	
None of the above	−	2	
Challenges with responsibility when something goes wrong	70	−	
Dependence on AI / reduced ability to diagnose without AI over time	63	−	
Extra work, e.g., when AI and the clinician disagree, or in the case of false positive results	52	−	
Poorer patient relationships	38	−	
Being fully or partially replaced by AI in the future	36	−	
Increase in diagnostic errors	25	−	
Negative changes in work tasks or disruptions in workflow	19	−	
Increased costs for the department	6	−	
I trust that my health data are stored and used in a secure, lawful, and ethically responsible manner for training AI systems by:			
Public healthcare providers	78	86	0.184
Private healthcare providers	31	23	0.312
The research and university sector	71	72	1.000
Industry or commercial suppliers	8	11	0.613
None of the above	13	16	0.530
**My trust in an AI system increases if it is:**			
Developed in Norway	52	56	0.646
Developed in Europe	50	52	0.879
Developed in other parts of the world	9	9	1.000
Trained on Norwegian data	61	47	0.092
Trained on European data	34	46	0.163
Trained on data from other parts of the world	6	10	0.433
None of the above	19	21	0.849
*p < 0.05			

Patients’ primary concerns were clinicians’ potential overreliance on AI-CDSS, insufficient clinician understanding of these systems, limited access to physician consultation when desired, and unclear responsibility in the event of errors. In comparison, healthcare personnel reported higher concern regarding responsibility and accountability, overreliance on AI, deskilling, and that AI implementation could increase their workload.

Both groups expressed similar skepticism (p > 0.05) toward private healthcare providers and industry storing and utilizing health data in a lawful and ethical manner and showed a preference for AI-CDSS developed in Norway or Europe using Norwegian or European data.

**Fig. 1:**
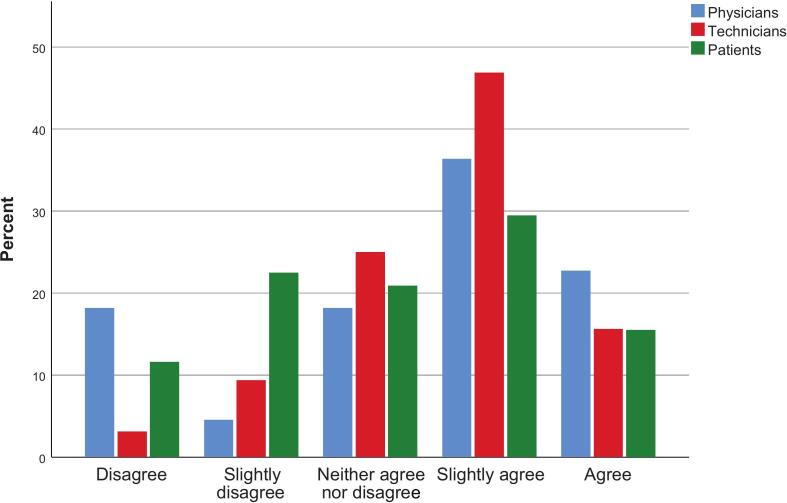
Response distributions for the statement “I would have equal trust in the decision from the AI-CDSS as from a physician”. Based on scenario text (cf. Methods section).

The answer distribution for the scenario described above is illustrated in Fig. 1. There was no difference between groups (p = 0.133), although technicians tended toward higher trust in the decision when compared with patients (p = 0.056).

**Fig. 2:**
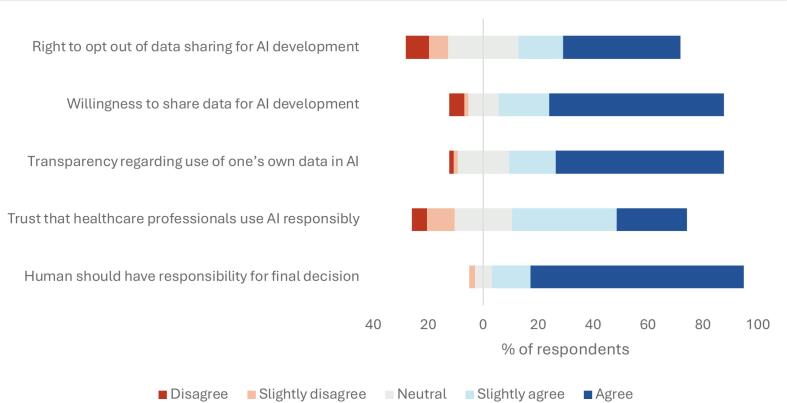
Short-form presentation of selected Likert items. The top four items were only answered by patients. The bottom item concerning whether healthcare professionals should always retain final decision-making responsibility was answered by all respondents and is shown pooled, as responses did not differ between groups (p = 0.688).

Remaining Likert items presented only to patients are presented in Fig. 2. The bottom item in figure 2 shows the response distribution to the question “It is important to me that healthcare personnel retain the final decision-making responsibility when AI is used”, which was posed to all respondents. Both groups agreed with this statement, and their results were pooled due to being largely identical (median 5, IQR 1, p = 0.688).

Free-text responses did not identify major concerns beyond those captured by the structured items. Respondents in both groups frequently emphasized that trust in AI-CDSS depends on documented clinical performance, high-quality and representative training data, and agreement with clinician judgment. Transparency, clear responsibility and the ability to understand how the system reaches its conclusions were also highlighted as important factors. Healthcare personnel further noted that prior experience with poorly performing AI systems in clinical practice, including challenges related to implementation, reduced their trust in AI-CDSS more broadly.

## 4 Discussion

We investigated perceptions of AI-CDSS among healthcare personnel and patients examined with clinical neurophysiological methods. In the following, we interpret the principal results considering existing literature.

### 4.1 Expectations

Both patients and healthcare personnel expected that implementation AI-CDSS would lead to more accessible healthcare, with shorter waiting times and increased efficacy, in keeping with existing literature (Amann et al., 2023, Giavina-Bianchi et al., 2024, Young et al., 2021). Patients also expected AI-CDSS to be helpful in diagnosing disease more accurately. In contrast, the response from healthcare personnel was more mixed, not necessarily agreeing that AI-CDSS would improve their abilities. This difference may reflect patients’ greater optimism toward technological solutions to tasks that may be somewhat abstract to a non-professional, while healthcare personnels’ responses could be based on greater familiarity with diagnostic uncertainty, contextual factors and potential sources of error. A larger proportion of healthcare personnel expected more fair and equitable services across regions instead, including Norway’s rural areas. Healthcare personnel are likely to have greater awareness of existing regional disparities in access to specialist expertise and services within their field, which may explain why they more readily recognize the potential of AI-CDSS to promote more accessible and equitable care (Ayorinde et al., 2024, Balakrishnan et al., 2025, NOU4, 2023).

### 4.2 Concerns

Prior research has shown that these concerns related to AI-CDSS are shared across medical fields (Amann et al., 2023, Kostick-Quenet et al., 2024, Steerling et al., 2023, Stewart et al., 2024, Young et al., 2021). Our findings support previous findings that some of the most pressing concerns are generally related to diagnostic accuracy, unease with AI-supported diagnoses, deterioration of the doctor-patient relationship, responsibility and accountability, overreliance and deskilling. Several of these concerns are interrelated and will be discussed together.

*Diagnostic accuracy.* First, diagnostic accuracy and unease with AI-supported diagnoses is a difficult concern to address. On the one hand, the patient group expected higher diagnostic accuracy and showed a positive attitude toward usage of AI-CDSS. On the other hand, it has been well-documented that both patients and clinicians are generally uncomfortable with AI-driven diagnoses, even when highly accurate, and more so for serious disease or if the computer program cannot “explain” how the diagnosis was made (Amann et al., 2023, Ayorinde et al., 2024, Khullar et al., 2022, Wang et al., 2023, Young et al., 2021). We found that clinicians did not expect an increase in diagnostic accuracy. This is reinforced by evidence that human decision-making is typically preferred over AI-CDSS, even when less accurate (Fritsch et al., 2022, Longoni et al., 2019, Yokoi et al., 2021, York et al., 2020). Negative expectations and preference for human decision-making may translate into skepticism toward AI-CDSS in clinical practice, which is problematic given that lack of acceptance by clinical teams is one of the most significant threats to successful adoption and implementation (Rojas et al., 2022, Severinsen et al., 2025, Silsand et al., 2024).

*Responsibility and doctor-patient relationship.* Second, also related to the fact that patients prefer a human decision-maker, is the fact that responsibility and accountability are of high concern to both patients and healthcare personnel. It is clear from both our findings and previous work (Amann et al., 2023, Fritsch et al., 2022, Jones et al., 2023, Lennartz et al., 2021, Longoni et al., 2019, Stewart et al., 2024, Wang et al., 2023, Yang et al., 2024, Yokoi et al., 2021, Yoo et al., 2023, York et al., 2020, Young et al., 2021) that patients require assurance that the final decision, and ultimately the responsibility, needs to rest with a human. This is solved through existing and developing legal frameworks in every country, but transparent communication with patients regarding decision-making processes and accountability remains essential to preserve trust and sustain the doctor–patient relationship. As AI-CDSS may facilitate task shifting, attention to communication practices and appropriate support for neurophysiology technicians when conveying results may also be important.

In addition, human-centered design is necessary to avoid deterioration of the doctor–patient relationship through so-called *technology-induced dehumanization* (Amann et al., 2023, Esmaeilzadeh, 2020, Giavina-Bianchi et al., 2024, Khullar et al., 2022, PewResearchCenter, 2023, Sauerbrei et al., 2023, Tun et al., 2025, Wang et al., 2023, Young et al., 2021). The goal is that interaction with the AI-CDSS should aid and augment the healthcare professional (Amershi et al., 2019). However, implementation of AI-CDSS may prove difficult even with high diagnostic accuracy: In our scenario where the AI-CDSS was defined as non-inferior to physician decision-making, a decision made by the nurse technician with the help of AI-CDSS was distrusted by more than 20% of physicians, with another 20% uncertain. Without interviewing the physicians in question, it is difficult to ascertain the underlying reasons for this apprehension, whether it reflects concerns about task-shifting and loss of professional control, uncertainty regarding responsibility and accountability, or skepticism toward the reliability and contextual understanding of AI-CDSS. In addition, as the scenario combines multiple elements, the specific driver of reduced trust cannot be determined, and responses likely reflect their combined effect in a plausible clinical workflow. Regardless of explanation, this finding underscores that even highly accurate AI-CDSS may face resistance when they alter established clinical roles and decision-making hierarchies.

The scenario was based on a relatively benign and standardized condition that could plausibly be implemented within a few years. Trust may differ in the context of more complex or severe diagnoses, where the consequences of diagnostic error are greater and the need for physician involvement is higher (Khullar et al., 2022). Finally, as illustrated by the scenario-based question, when no definitive diagnosis is established by the technician and AI-CDSS but symptoms persist, clearly defined follow-up pathways from referring physicians or institutions become particularly important to maintain patient trust.

*Deskilling and overreliance on AI.* Third is the concern of how to manage overreliance on AI, and the expected negative feedback loop between dependency on AI support, deskilling, and its effect on professional environments and training. This fear of skill atrophy has been replicated across multiple studies (Amann et al., 2023, Esmaeilzadeh, 2024, Giavina-Bianchi et al., 2024, Perivolaris et al., 2024, Stewart et al., 2024, Yang et al., 2024, Yoo et al., 2023, Young et al., 2021), and for good reasons. Patients worry that the human decision-maker they rely on may become more error-prone and reluctant to challenge the AI-CDSS’ diagnoses when they ought to, while healthcare personnel are naturally concerned that their diagnostic competence may erode over time, even as their professional responsibility remains unchanged. One argument for implementing AI-CDSS in clinical practice in general is that simpler cases could be filtered out, allowing physicians to focus on more complex cases. However, this could both create more niche super-specialists instead of generalists, and reduce exposure to the entire range of patients, which is paramount to becoming a well-rounded physician that understands the spectrum between normal and abnormal findings. It could potentially also alter professional roles and be cause for potential interprofessional tensions, with clinical neurophysiologists assuming greater responsibility for oversight, validation, and quality assurance of AI-CDSS, as well as supporting other clinicians who rely on these systems. There is no simple solution to the challenge of dependency on AI-support and deskilling. Mitigating these risks will likely require careful workflow integration, continuous training, and active involvement of healthcare personnel in the design and implementation of AI-CDSS to ensure that clinical expertise is preserved.

### 4.3 Attitudes and trust

Although we found an overall positive attitude toward the use of AI-CDSS in clinical neurophysiology in both groups, responses to the concrete clinical scenario revealed more reserved levels of trust. When asked to consider a specific, non-abstract use case in which AI-CDSS directly influenced clinical decision-making and the need for physician consultation, respondents expressed greater caution than signaled by their general attitudes. This gives some cause for pause, especially when considering that our sample is unlikely to be entirely representative, with respondents showing a high degree of interest in AI. Our results echo previous findings that proven, high diagnostic accuracy is unlikely to be sufficient to overcome concerns and skepticism toward AI-CDSS in clinical neurophysiology, other assurances are also needed, such as transparency and AI-literacy. AI literacy is generally lacking among healthcare personnel (Castagno and Khalifa, 2020, Oh et al., 2019, Scheetz et al., 2021), but can be addressed through education. It should begin at the undergraduate level and continue throughout professional practice to ensure end-users have sufficient general proficiency (Al Hadithy et al., 2023, Chan and Zary, 2019, Esmaeilzadeh, 2024, Grunhut et al., 2021, Kundu, 2021, Mousavi Baigi et al., 2023).

Furthermore, factors that promote trust often inversely correspond to those that reduce it. One example is transparency *versus* black-box models (Amiri et al., 2024, Ayorinde et al., 2024, Kostick-Quenet et al., 2024, Rojas et al., 2023, Shevtsova et al., 2024, Shick et al., 2024, Steerling et al., 2023, Tun et al., 2025, Wang et al., 2023), another concerns systems built and implemented with input from patients and end-users, as opposed to purely data-driven approaches that give limited consideration to practical feasibility, such as integration with existing, often inadequate hospital IT infrastructure (Kostick-Quenet et al., 2024, Perivolaris et al., 2024, Stewart et al., 2024, Wang et al., 2023). At the same time, our findings also highlight a set of necessary (but not sufficient) conditions for patient trust. Patients strongly agreed that they should be able to determine whether their data have been used to train AI models, and that they should have the option to opt out. Both conditions presuppose access to timely and adequately detailed information on the data used for training, which may reduce the speed of AI-development significantly. Importantly, patients also expressed a high degree of institutional trust in the public healthcare and university sectors, and the vast majority indicated willingness for their personal data to be used for AI development that could benefit themselves and other patients. This aligns with prior literature demonstrating that patients generally desire control over their data, but, given sufficient assurances of data security and privacy, are willing to make them available for AI training (Aggarwal et al., 2021, Beets et al., 2023, Fritsch et al., 2022, Yang et al., 2024, Young et al., 2021).

### 4.4 Explainability and transparency

Many of the discussed themes tie into the type of AI-system used, and whether it can be readily understood. For instance, skill atrophy and inherent skepticism toward AI-based diagnoses go hand in hand when the systems lack transparency. In our survey, patients expressed concern that healthcare personnel may not have sufficient understanding of how the AI-CDSS work, often related to the notion of “black boxes”: Algorithms that are either inaccessible or so impenetrably complex that humans cannot meaningfully interpret them. Interpretability concepts such as explainability and transparency are more difficult to operationalize and are often used interchangeably. For our purposes we will define explainability as “AI systems that can explain their rationale to a human user and convey an understanding of how they will behave in the future” (Gunning and Aha, 2019), or in other words, the “why” behind the AI-CDSS’ decision. Transparency refers more specifically to documentation and openness regarding data inputs, algorithmic logic, and methods of evaluation and validation (IBM, 2026), or “how” the model works. Black-box systems introduce an aura of unpredictability and represent a clear barrier to trust (Esmaeilzadeh, 2024), which has contributed to a considerable push toward more explainable AI (Labkoff et al., 2024, Sun et al., 2025). This emphasis is reflected in key regulatory frameworks, including the EU AI Act, the US AI Bill of Rights, and the US Executive Order on safe, secure, and trustworthy AI, all of which stress the need for transparency, adequate AI literacy among healthcare personnel, and that the patients should be able to make an informed decision on whether or not to opt out.

Challenges concerning transparency and explainability are not easily resolved. Model accuracy and explainability appear to be at least partly inversely related (Gunning and Aha, 2019), and meaningful understanding by healthcare personnel would require both foundational AI literacy and tool-specific documentation tailored to clinical users. Such educational efforts are also resource-intensive, which stands in tension with one of the core motivations for AI-CDSS adoption: improving healthcare efficiency. At the same time, utilizing model interpretability tools such as SHAP (Lundberg and Lee, 2017) or LIME (Ribeiro et al., 2016) to explain feature weighting in complex models is unlikely to be meaningful for either patients or clinicians. Instead, clinicians require insight into clinically relevant model features that align with established evidence-based practice (Tonekaboni et al., 2019). This need is underscored by examples of AI systems learning spurious, clinically irrelevant correlations, such as classifying malignant skin lesions based on the presence of a ruler in the image (Esteva et al., 2017), predicting hip fractures based on “urgent” metadata labels (Badgeley et al., 2019), or identifying pneumonia based on the type of X-ray equipment used (Zech et al., 2018). A middle ground exists somewhere. Clinicians are not expected to understand the internal workings of MRI scanners or the software used to produce the image and correct artefacts. Yet neither patients nor the medical community question the clinical legitimacy of such technologies or debate shared responsibility between clinicians, hospitals, and commercial vendors. It may at least partly come down to the different associations and mental imagery regarding what AI really is, again reinforcing the importance of AI literacy and providing users with the right amount of relevant information about the AI-CDSS in question.

### 4.5 Research and development

4.5

These findings are particularly relevant to clinical neurophysiology for two interconnected reasons. First, the field sits on a rapidly growing treasure trove of high-resolution data. Researchers seek to use these data, and patients are largely willing to share them. However, navigating the jungle of regulatory frameworks governing the development, validation, and implementation of AI-CDSS is a daunting and resource-intensive task that can stop even the most promising project. Second, patients distrust industry partners’ and commercial suppliers’ ability to handle data in a secure, lawful and ethical manner. Yet meaningful AI-CDSS development is unlikely without industry collaboration. Academic researchers may develop functioning models, but typically lack the time, resources, and regulatory expertise required to translate these into CE-marked or FDA-approved medical devices. Consequently, building and maintaining patient trust should be viewed as a shared responsibility between public institutions, researchers, and industry partners.

### 4.6 Limitations

Some limitations should be acknowledged. First, some non-response bias may be present, potentially reflecting either limited interest in AI or more negative attitudes toward AI-based solutions. The use of SMS-distributed questionnaires may also have introduced selection bias by excluding patient groups with lower digital engagement. Together, these factors may have led to an overrepresentation of respondents who are more familiar with or positively inclined toward AI, potentially inflating estimates of acceptance and expected benefits. However, the range of responses observed, including some clearly critical perspectives, suggests that the sample was not entirely skewed toward AI-positive respondents. Future studies should aim to include underrepresented groups, particularly those with lower digital engagement, through alternative recruitment strategies such as in-person approaches or adapted survey formats. Second, trust in and acceptance of AI may be influenced by demographic and contextual factors such as age, sex, education level, prior experience with technology, technical affinity, and healthcare utilization (Fritsch et al., 2022, Giavina-Bianchi et al., 2024, Young et al., 2021). The sample size did not permit reliable adjustment for these factors, and exploratory analyses were not pursued to avoid overinterpretation in a limited sample. However, the primary aim of the study was exploratory and descriptive, rather than estimating population-level effects. Third, while we identified subgroups such as the 15–20% of physicians who expressed low trust in technician’s decision making when supported by an AI-CDSS, the present design does not allow for deeper exploration of the underlying reasons for this distrust. Fourth, the healthcare personnel group was heterogeneous and attitudes toward AI-CDSS may differ between subgroups. The sample size did not permit reliable subgroup analyses, and this should be explored in future studies. Fifth, although analyses were exploratory and items reflected distinct constructs, multiple comparisons could increase the risk of type I error and significant findings should therefore be interpreted with caution. Finally, the study was conducted in Norway, which has a universal and publicly tax-based healthcare system characterized by high levels of institutional trust. These contextual factors may limit the generalizability of the findings to settings with different levels of trust, healthcare organization, or greater reliance on out-of-pocket financing.

## 5 Conclusions

In conclusion, while overall attitudes toward AI-CDSS were positive, our findings highlight that trust, acceptance, and successful implementation depend on more than just diagnostic accuracy. Both patients and clinicians have persisting concerns regarding responsibility, transparency, overreliance and workflow integration, de-skilling and doctor-patient relationships. Skepticism toward AI-CDSS is most pronounced when AI-CDSS directly influence clinical decision-making and access to physician consultation. Patients expressed high institutional trust and willingness to share data for AI development under conditions of transparency, data security and –privacy, and an out-opt mechanism. Clinical neurophysiology is well positioned to benefit from AI-CDSS, provided a focus on realistic implementation and human-centered design.

## Author contributions

**Ø. Dunker:** Conceptualization, methodology, formal analysis, writing – original draft, review and editing, project administration, funding acquisition. **L.O Bergsjø:** Conceptualization, methodology, writing – review and editing. **G.H. Severinsen:** Conceptualization, methodology, writing – review and editing. **H. Glette:** Conceptualization, review and editing. **K.B. Nilsen:** Conceptualization, methodology, writing – review and editing, project administration, funding acquisition.

## Ethical statement

The collection and processing of data was approved by the local data protection officer at Oslo University Hospital, in accordance with relevant EU and National laws. All participants gave informed consent and the data collected was anonymous. Further ethical approval by the regional ethics committee was not required.

## Declaration of competing interest

The authors declare that they have no known competing financial interests or personal relationships that could have appeared to influence the work reported in this paper.
